# Cardiac Point of Care Ultrasound (POCUS) Used to Diagnose Infective Endocarditis Following Multiple Negative Echocardiograms

**DOI:** 10.24908/pocusj.v10i01.17855

**Published:** 2025-04-15

**Authors:** Adriano Sanjuan, Daniel S Brenner, Heather Andrade, Alyson Bundy, Philip Clapham, Nathan Markus, Irina K Hariri, Edwin Jackson

**Affiliations:** 1 Internal Medicine Residency Program, Indiana University School of Medicine, Indianapolis, IN, USA; 2 Department of Emergency Medicine, Indiana University School of Medicine, Indianapolis, IN, USA; 3 Internal Medicine-Pediatrics Residency Program, Indiana University School of Medicine, Indianapolis, IN, USA; 4 Division of Pulmonary and Critical Care Medicine, Indiana University School of Medicine, Indianapolis, IN, USA; 5 Division of Infectious Disease, Indiana University School of Medicine, Indianapolis, IN, USA

**Keywords:** infective endocarditis, POCUS, transthoracic ecocardiography (TTE), aortic valve endocarditis

## Abstract

Infective endocarditis (IE) is a life-threatening condition often diagnosed using the modified Duke's criteria, including bacteremia and pathognomonic echocardiographic findings. However, up to 30% of cases yield inconclusive results with transthoracic echocardiograms (TTE) or transesophageal echocardiograms (TEE). We present a case of a 68-year-old man with methicillin-susceptible *Staphylococcus aureus* (MSSA) bacteremia and recurrent fevers, in which multiple echocardiograms failed to detect valvular vegetations. However, an advanced cardiac point of care ultrasound (POCUS) examination identified a vegetation on the aortic valve, later confirmed by TTE and TEE. Although generalization is limited due to operator expertise and patient characteristics, this case demonstrates the utility of advanced cardiac POCUS in diagnosing IE in critically ill patients with negative initial echocardiograms. Incorporating advanced cardiac POCUS into routine diagnostic workflows may improve diagnostic accuracy and patient outcomes. Increasing use of advanced cardiac POCUS also highlights the importance of expanding proficiency among intensivists.

## Introduction

Infective aortic valve endocarditis (AVE) is a potentially fatal condition caused by infection of the aortic valve, predominantly by methicillin-susceptible *Staphylococcus aureus* (MSSA) or *Streptococcus* species. AVE typically progresses through several stages. It begins with bacteremia and is followed by the formation of valvular vegetations, which can lead to valvular destruction, heart failure, embolic events, systemic infection, and ultimately death [1–3]. Early and accurate diagnosis is crucial, as it can significantly impact patient management [4]. In clinical practice, echocardiography is an essential tool for diagnosing endocarditis, as endorsed by guidelines from the Infectious Diseases Society of America, the American Heart Association, and the European Society of Cardiology [2,5,6]. These guidelines recommend a transthoracic echocardiogram (TTE) as the initial imaging modality due to its non-invasive nature and widespread availability. However, when vegetations are not found on the TTE and clinical suspicion remains high, a transesophageal echocardiogram (TEE) is recommended due to its superior diagnostic sensitivity and specificity in the identification of valvular vegetations, abscess, and occult complications of IE. Serial echocardiograms are necessary when the clinical suspicion for endocarditis remains high despite negative initial TTE and TEE results. The optimal timing for subsequent echocardiograms is debated, but both the American Heart Association and the European Society of Cardiology recommend follow-up exams within one week of negative initial TTE or TEE studies if clinical concern persists [2,5,6]. No clear guidelines exist on the time of repeating imaging if the follow-up echocardiogram is also negative. With clinicians' increasing comfort and expertise with POCUS—particularly advanced cardiac POCUS—and its ability to evaluate the aortic, mitral, and tricuspid valves with standard views [7], these exams can be a viable alternative to repeated echocardiograms in clinical settings. Several studies validate the importance of daily POCUS as a complement to the clinical assessment, with work by Palmero et al. highlighting the effectiveness of cardiac POCUS in detecting infective endocarditis (IE) among patients with bacteremia [8–11]. Our case report reinforces this approach, demonstrating that daily advanced cardiac POCUS can effectively identify valvular vegetation when initial TTE and TEE exams have yielded inconclusive results. Daily advanced cardiac POCUS examinations could be considered in cases of suspected endocarditis, particularly when there is high clinical suspicion and initial and repeat echocardiographic examinations are negative. This strategy may optimize healthcare resource utilization and reduce costs, while simultaneously improving the diagnostic accuracy for IE.

## Case Report

This case report adheres to the CARE Guidelines for standardized clinical case reporting [12]. We present a case of a 68-year-old African American man with a history of housing insecurity, chronic obstructive pulmonary disease, chronic hypoxic respiratory failure, heart failure with mildly reduced ejection fraction, chronic kidney disease, hypertension, opioid use disorder, schizophrenia, and mild mitral, tricuspid, and aortic valve insufficiency. He was admitted to the Medical Intensive Care Unit (MICU) after presenting to the emergency department due to multiple traumatic rib fractures sustained after a non-witnessed fall. At presentation, he was not able to provide patient history, and his initial vital signs showed blood pressure at 170/117 mmHg, heart rate of 94 bpm, temperature of 97.8 degrees Fahrenheit, respiratory rate at 17 rpm, and peripheral oxygen saturation at 94% on 4 liters of supplemental oxygen via nasal cannula. The physical exam was remarkable for altered mental status, apparent tongue swelling, tenderness to palpation on the right chest, and abrasions over the left thigh. His course was complicated by progressive hypoxic respiratory failure necessitating intubation and mechanical ventilation. Following intubation, he began to experience persistent fevers, worsening hypoxia, and purulent respiratory secretions. An initial computed tomography (CT) of the chest without contrast revealed multifocal cavitary pneumonia primarily in the right upper lobe, raising concerns for tuberculosis. However, subsequent negative acid-fast bacilli smears and bronchoalveolar lavages excluded tuberculosis, and instead identified infections with MSSA and *Streptococcus agalactiae*. Within 24 hours of the respiratory cultures becoming positive, blood cultures also returned positive for MSSA. He subsequently developed septic shock, acute respiratory distress syndrome, and severe acute kidney injury. Given his initial bacteremia, a TTE was performed to evaluate for IE. This revealed no valvular vegetations but showed elevated right ventricular systolic pressure and mild chronic valvular regurgitation affecting the mitral, tricuspid, and aortic valves, consistent with his known history (Video S1). Due to the MSSA and concerns for IE, a TEE was performed two days later. This revealed a reduced left ventricular ejection fraction, mild concentric left ventricular hypertrophy, and left atrial dilatation, but no valvular vegetations (Video S2).

Over the next seven days, he remained critically ill with persistent fevers, and sputum cultures consistently showed MSSA alongside newly detected *Stenotrophomonas maltophilia*. At the same time, his blood cultures cleared, and a repeat chest CT without contrast showed pulmonary cavitation. A few days later, his fever and hypotension resolved, and he was extubated, allowing for transfer from the MICU to the general medical ward. Approximately one week after transfer, subsequent imaging revealed atelectasis and the known pulmonary cavitary lesion. Despite negative blood culture results, the patient's respiratory status continued to decline, and he was readmitted to the MICU due to progressive respiratory failure, necessitating urgent intervention with endotracheal intubation and bronchoscopy, which confirmed ongoing MSSA pneumonia.

Due to persistent fevers, an extensive secondary fever workup was pursued and ruled out potential causes, including retroperitoneal and cerebral hemorrhages, deep vein thrombosis, sinusitis, meningitis, and autoimmune disorders.

Despite serial negative blood cultures, the fevers persisted, raising continued concerns for IE. This prompted the MICU team—comprising of a first-year Internal Medicine resident, a Pulmonary and Critical Care Medicine fellow, and supervised by a Pulmonary and Critical Care Medicine faculty member who is both a Registered Diagnostic Cardiac Sonographer and a Diplomate of the National Board of Echocardiography in Critical Care Echocardiography—to conduct daily advanced cardiac POCUS exams.

**Figure 1.**
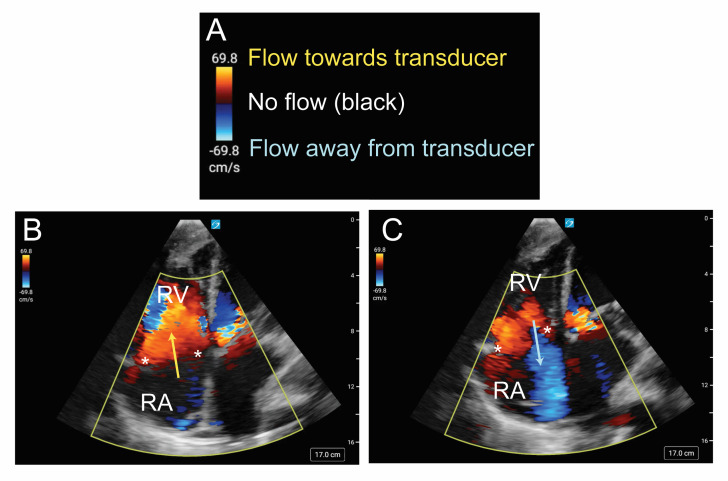
Advanced cardiac point of care ultrasound (POCUS) illustrating tricuspid regurgitation.

The exams were performed using a SonoSite PX ultrasound system (Fujifilm; Bothwell, WA) equipped with a phased array transducer (5–1 MHz). This protocol, standard at our institution, included obtaining parasternal long-axis, right ventricular inflow and outflow, parasternal short-axis, and apical 4- and 5-chamber views. Color flow Doppler was applied specifically over the mitral, aortic, and tricuspid valves to assess for new valvular abnormalities or vegetations (Figure 1, Video S3). In experienced hands, the entire protocol can be completed in under five minutes.

**Figure 2.**
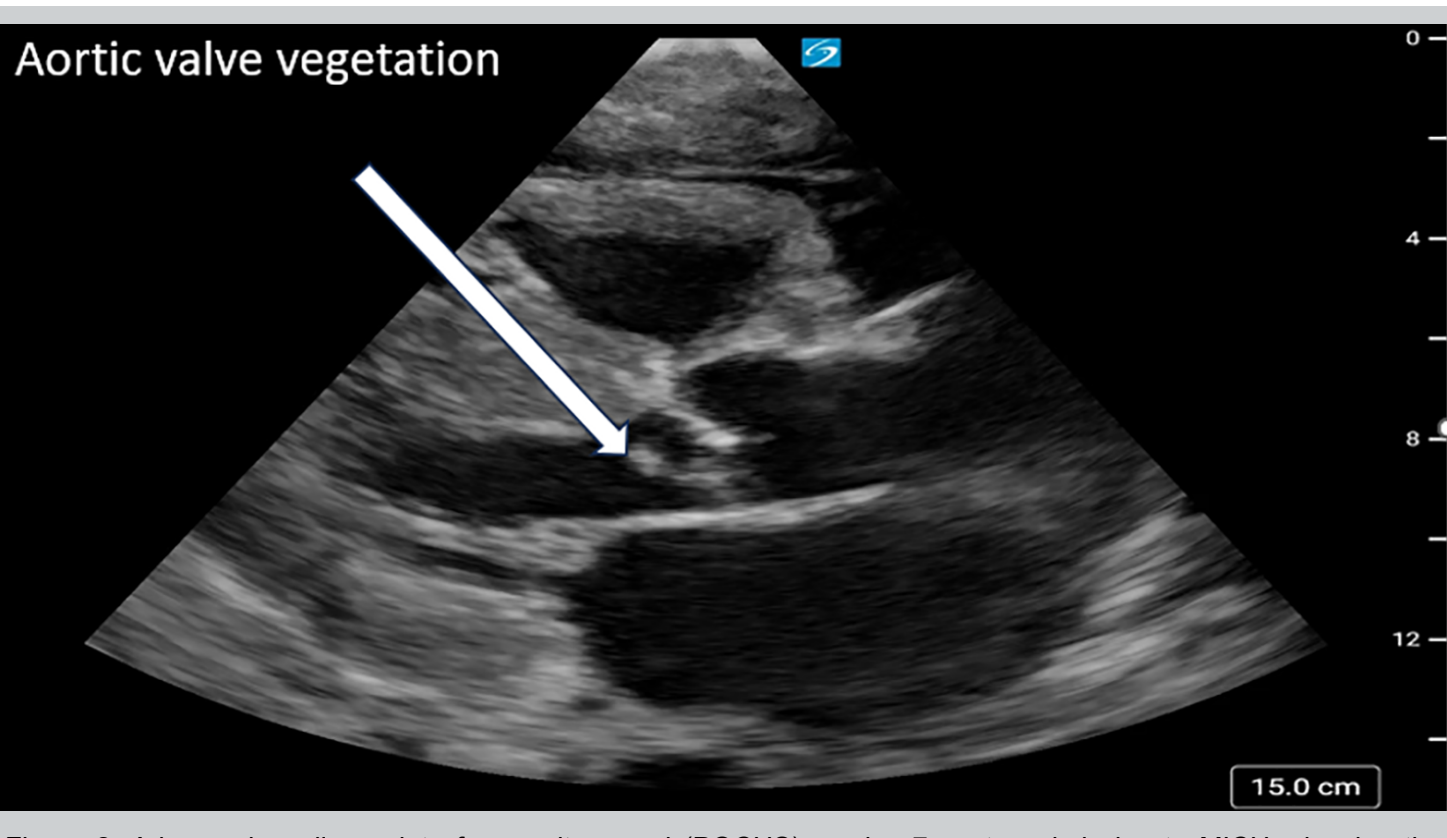
Advanced cardiac point of care ultrasound (POCUS) on day 7 post-readmission to MICU, showing the parasternal long axis view demonstrating an aortic valve vegetation.

**Figure 3.**
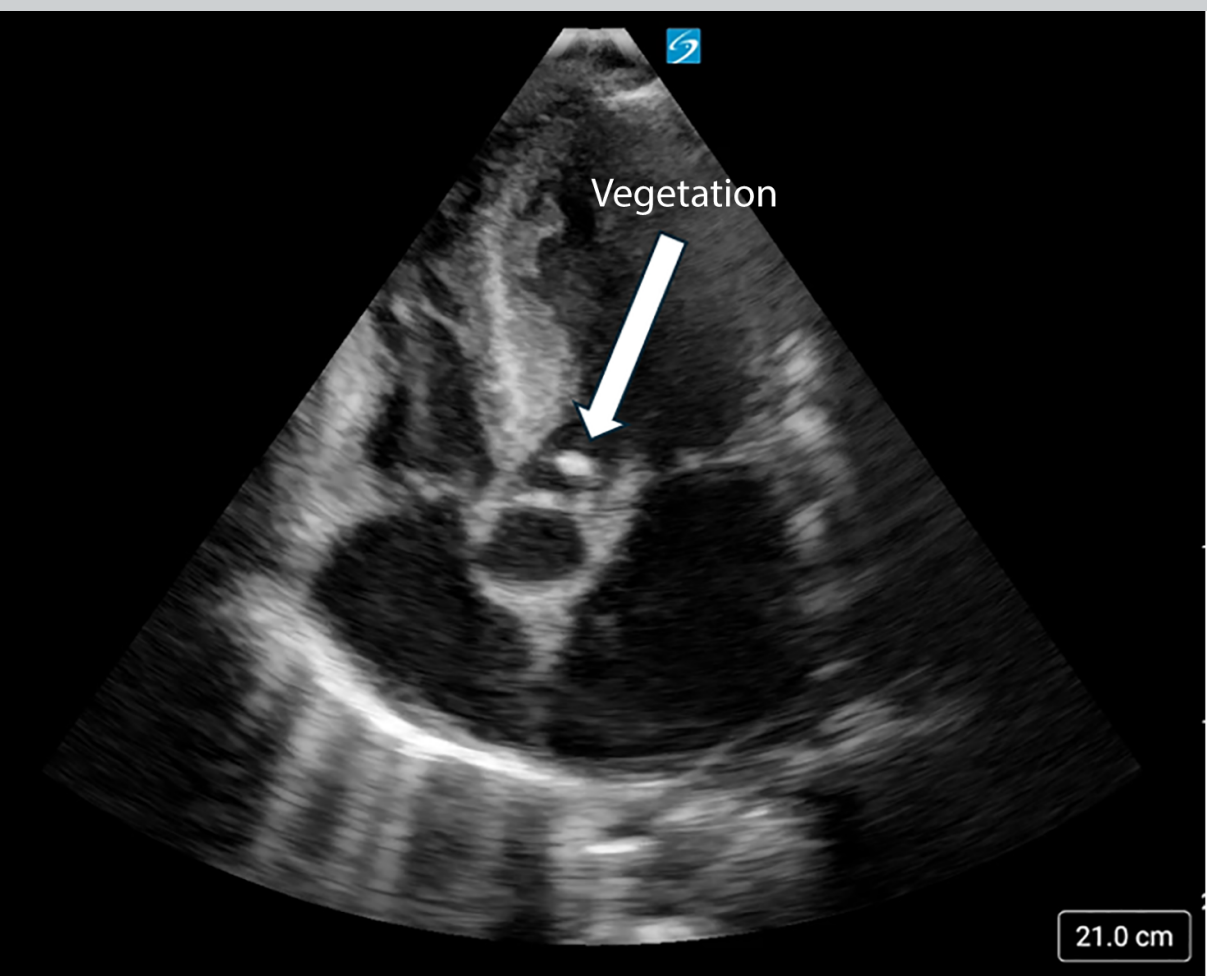
Advanced cardiac point of care ultrasound (POCUS), apical 5-chamber view with a vegetation on the ventricular side of the aortic valve.

**Figure 4.**
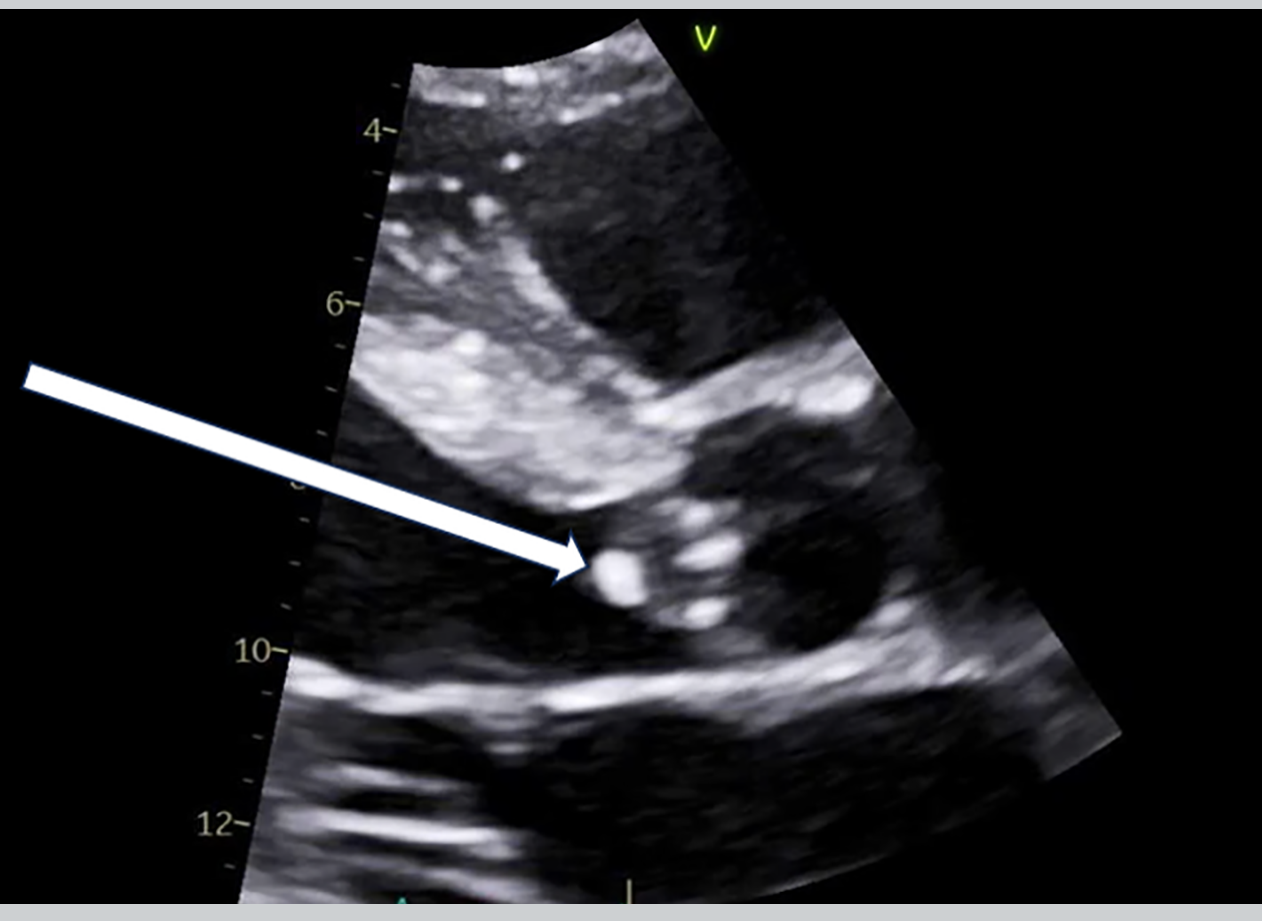
Transthoracic echocardiography (TTE) parasternal long axis zoomed view at the left ventricular outflow tract confirming the left coronary cusp vegetation pointed by the arrow.

**Figure 5.**
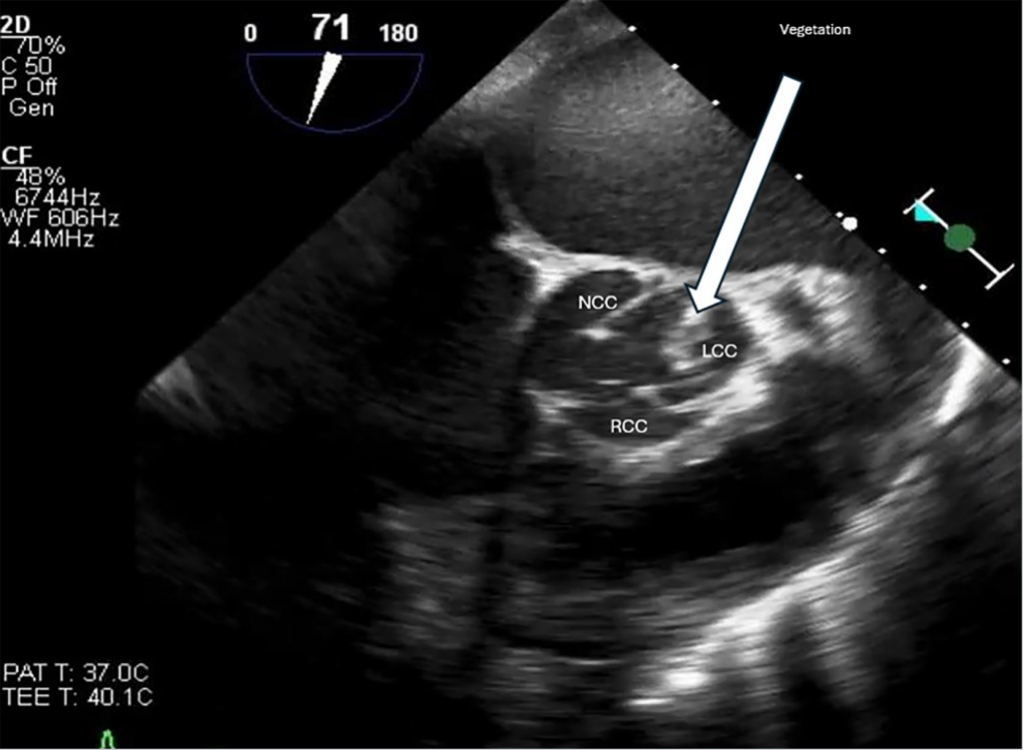
Transesophageal echocardiography (TEE) on day 10 post-readmission to medical intensive care unit (MICU) with mid esophageal short axis view showing vegetation on the left coronary cusp (LCC). LCC, left coronary cusp; RCC, right coronary cusp; NCC, non-coronary cusp.

Seven days after his second MICU admission, an advanced cardiac POCUS examination identified a 0.8 cm vegetation on the ventricular side of the left aortic valve cusp with associated aortic regurgitation (Figures 2 and 3). This finding prompted a subsequent confirmatory echocardiography with repeat TTEs and TEEs, which confirmed a large left coronary cusp vegetation associated with severe aortic valve regurgitation (Figures 4 and 5). The TTEs on this patient were performed by cardiac sonographers, while the TEEs were performed by the cardiology fellow and cardiology attending. Both exams were interpreted by a cardiologist. Unfortunately, following the discovery of AVE, the patient continued to decompensate, developing septic emboli and a worsening mental status, which precluded surgical intervention. Given the severity of his condition and the potential complications associated with anticoagulation, surgical options became less feasible. After thorough discussions with his family, a decision was made to shift from aggressive curative efforts to comfort-oriented care. The patient underwent terminal extubation after enduring a challenging 48-day hospital stay.

## Discussion

IE is a potentially fatal condition caused by infection of the heart valves or cardiac chambers; usually through MSSA or *Streptococcus* bacteria [1–3]. Recognizing new vegetations on an echocardiogram is an integral component of diagnosing IE as it constitutes a major criterion in the modified Duke criteria (Video S4, Table S5) [3]. Valvular vegetations associated with IE typically appear on the flow side of the valves: the atrial side of the mitral and tricuspid valves and the ventricular side of the aortic and pulmonary valves, which can lead to acute valvular regurgitation rather than stenosis [6]. In this case, advanced cardiac POCUS demonstrated a mobile density on the ventricular side of the aortic valve, in addition to new onset severe aortic regurgitation not seen on prior TTE, TEE, or advanced cardiac exams. Furthermore, our patient had only one positive blood culture upon his first admission to the MICU, which cleared and remained negative for the rest of his hospital course.

This case had an atypical presentation, both clinically and echocardiographically, and illustrated the role of advanced cardiac POCUS in the detection and management of IE. It exemplified the diagnostic challenges associated with this disease. Despite adherence to guideline-recommended imaging protocols by the Infectious Diseases Society of America , American Heart Association, and European Society of Cardiology, which recommend the use of TTE as the initial diagnostic tool followed by TEE, our patient's condition remained unresolved until the application of daily advanced cardiac POCUS [2,5]. This highlights the need for novel imaging strategies in cases where conventional methods are insufficient.

Daily advanced cardiac POCUS offers several advantages, including its bedside availability, cost-effectiveness, and ability to provide real-time diagnostic information [7–9]. These characteristics allow POCUS to provide dynamic assessment and fast clinical decision-making, which is crucial in the MICU setting. Additionally, the ability to easily perform repeated daily examinations with minimal disruption to the patient adds a layer of continuous monitoring that is a key component in managing such complex cases [10]. The protocol we used includes the parasternal long axis and right ventricular inflow and outflow views, which can identify tricuspid and pulmonic regurgitation. In the right ventricular inflow view, the anterior and septal leaflets of the tricuspid valve are visible. The parasternal long axis view also allows for the identification of mitral and aortic regurgitation, as well as visualization of the anterior and posterior mitral valve leaflets and the right and non-coronary cusps of the aortic valve. We then transitioned to the parasternal short axis view at the aortic and mitral valve levels (base) to further evaluate the tricuspid valve, mitral valve, and all three cusps of the aortic valve, including the left coronary cusp, which is often not visible in the parasternal long axis view. Next, we moved to the apical 4-chamber view, providing another perspective of the mitral and tricuspid valves. This view facilitated the evaluation of valvular regurgitation and a detailed examination of the septal, posterior, and anterior tricuspid leaflets with slight probe manipulation. Finally, the aortic valve was reassessed in the apical 5-chamber view (Video S6). While advanced cardiac POCUS is a promising tool and provides significant advantages, it is not exempt from limitations. Its accuracy is operator-dependent and requires training to be executed in the most reliable way. Variability in diagnostic accuracy based on the operator's experience raises the need for structured training programs and certification for clinicians using POCUS [11,13,14]. Additionally, the proficiency required for advanced cardiac POCUS is not universally possessed among intensivists, which could limit its widespread applicability in many intensive care settings. Expanding formal training and credentialing initiatives will be essential to overcome these challenges and to maximize the utility of advanced cardiac POCUS in clinical practice [15]. Moreover, we recognize the need for caution when interpreting our findings, as this study represented a single case report. As such, generalizability may be limited by the study design, the specific POCUS operator, and patient characteristics.

At our institution, the daily advanced cardiac POCUS exam includes the parasternal long axis, right ventricular inflow/outflow, parasternal short axis, and apical 4-and 5-chamber views. This expanded approach to the basic cardiac POCUS exam allows for comprehensive visualization and the application of color Doppler over all four cardiac valves, enabling the evaluation of new regurgitation. Although color Doppler is not incorporated into the basic cardiac POCUS workflow, this should not be considered a significant limitation to its use. A study by Hellmann et al. demonstrated that medical residents could achieve significant proficiency with color Doppler, with minimal formal training, which aligns with our experience [15].

Of the three additional views beyond the basic cardiac POCUS exam, the right ventricular inflow and the right ventricular outflow views are the easiest to achieve. They are obtained from the familiar parasternal long axis position but require knowledge of transducer movement known as “fanning” or “tilting.” For the right ventricular inflow view, the transducer tail is tilted towards the patient's left shoulder. For the right ventricular outflow view, the transducer tail is tilted towards the right hip to achieve a view of the tricuspid valve and pulmonic valve, respectively.

Lastly, the apical 5-chamber view is used in our daily advanced cardiac POCUS exam to evaluate for aortic valve regurgitation due to its favorable Doppler angle of the left ventricular outflow tract. This view is obtained by tilting the tail of the transducer towards the left hip while in the apical 4-chamber view.

Considering both its benefits and limitations, we advocate for the integration of daily advanced cardiac POCUS into routine clinical practice in the MICU, as an adjunct to, rather than a replacement of, a patient's physical exam or conventional sonographic examinations. By doing so, advanced cardiac POCUS can greatly improve both diagnostic accuracy and management of IE, especially in patients with atypical presentations, persistent symptoms, or when diagnosis is inconclusive by conventional imaging. This case portrays the limitations of TTE and TEE in such scenarios and points towards a potential use of daily advanced cardiac POCUS to serve as an extension of the physical examination.

The findings from our case support continued research on this topic and possibly reevaluation of current guidelines to incorporate daily advanced cardiac POCUS as a complementary tool in the diagnostic pathway for IE. Future studies should focus on validation of daily advanced cardiac POCUS and its ability to identify new valvular vegetations in MICU patients with possible IE when initial imaging techniques fail to make a definitive diagnosis. In patients diagnosed with IE using POCUS, outcomes such as mortality rate and timing from diagnosis to surgery should be assessed. By advancing our understanding and application of POCUS, we could improve patient outcomes and make more informed clinical decisions in complex cases of IE.












