# Supplementary material for: Cardiac Point of Care Ultrasound (POCUS) Used to Diagnose Infective Endocarditis Following Multiple Negative Echocardiograms
Source: POCUS J. 2025 Apr 15;10(1):72–7. doi: 10.24908/pocusj.v10i01.17855 (PMC12057459; doi:10.24908/pocusj.v10i01.17855)
Supplement: Supplementary file 5 [file pocusj-10-01-17855-s005.pdf]

Table S5. Definition of infective endocarditis according to the proposed modified Duke Criteria*

Definite infective endocarditis

Pathologic criteria: (1) microorganisms demonstrated by culture or histologic examination of a vegetation, a vegetation that has embolized, or an intracardiac abscess specimen; or

(2) Pathologic lesions; vegetation or intracardiac abscess confirmed by histologic examination showing active endocarditis

Clinical criteria: (1) 2 major criteria; or (2) 1 major criterion and 3 minor criteria; or (3) 5 minor criteria

Major clinical criteria

Blood culture positive for IE

Typical microorganisms consistent with IE from two separate blood cultures:

Viridans streptococci, *Streptococcus bovis*, HACEK group, *Staphylococcus aureus*; or

Community-acquired enterococci, in the absence of a primary focus; or

Microorganisms consistent with IE from persistently positive blood cultures, defined as follows:

At least 2 positive cultures of blood samples drawn > 12 h apart; or

All of 3 or a majority of $\geq$ 4 separate cultures of blood (with first and last sample drawn at least 1 h apart)

Single positive blood culture for *Coxiella burnetii* or antiphase I IgG antibody titer > 1 : 800

Evidence of endocardial involvement

Echocardiogram positive for IE (TEE recommended in patients with prosthetic valves, rated at least “possible IE”

by clinical criteria, or complicated IE [paravalvular abscess]; TTE as first test in other patients), defined as follows:

Oscillating intracardiac mass on valve or supporting structures, in the path of regurgitant jets, or on im- planted material in the absence of an alternative anatomic explanation; or

Abscess; or

New partial dehiscence of prosthetic valve

New valvular regurgitation (worsening or changing of pre-existing murmur not sufficient)

Minor clinical criteria

Predisposition to infectious endocarditis, predisposing heart condition or injection drug use

Fever, defined as temperature > 38°C

Vascular phenomena, major arterial emboli, septic pulmonary infarcts, mycotic aneurysm, intracranial hemorrhage, conjunctival hemorrhages, and Janeway’s lesions

Immunologic phenomena: glomerulonephritis, Osler's nodes, Roth's spots, and rheumatoid factor

Microbiological evidence: positive blood culture but does not meet a major criterion as noted above or serological evidence of active infection with an organism consistent with IE

Possible infective endocarditis

(1) One major criterion and 1 minor criterion; or (2) three minor criteria

Rejected infective endocarditis

(1) Firm alternate diagnosis explaining evidence of infective endocarditis; or

(2) Resolution of infective endocarditis syndrome with antibiotic therapy for <4 days; or

(3) No pathologic evidence of infective endocarditis at surgery or autopsy, with antibiotic therapy for <4 days; or

(4) Does not meet criteria for possible infective endocarditis, as above

* Reproduced with permission from Li et al, 2000 [3].
